# Supplementary material for: Decreasing Incidence and Mortality in Traumatic Brain Injury in Korea, 2008–2017: A Population-Based Longitudinal Study
Source: Int J Environ Res Public Health. 2020 Aug 26;17(17):6197. doi: 10.3390/ijerph17176197 (PMC7504501; doi:10.3390/ijerph17176197)
Supplement: Supplementary file 1 [file ijerph-17-06197-s001.pdf]

Table S1. Odds ratios from a comparison of the participants with traumatic brain injury (TBI) and individuals without TBI (age ≥70 years) from the general population.

Year	2008	2009	2010	2011	2012	2013	2014	2015	2016	2017
Odds ratio	26.9	25.3	20.1	17.8	14.2	12.6	10.7	8.6	6.5	4.4

Table S2. The crude incidence and mortality rates of participants in the 0–4 and 5–9 years groups.

Year	Age	2008	2009	2010	2011	2012	2013	2014	2015	2016	2017	*p* for Trend
Crude incidence rate	0–4	1818.8	1920.8	1921.5	1788.0	1738.5	1677.0	1487.5	1344.6	1353.4	1308.5	<0.001
	5–9	839.1	887.0	913.1	882.8	877.8	843.1	792.6	734.8	710.0	716.8	<0.001
Crude mortality rate	0–4	3.6	3.8	2.9	2.6	2.2	2.4	2.2	1.4	1.6	1.1	0.055
	5–9	1.6	1.9	1.4	1.1	1.1	0.7	0.6	0.3	0.7	0.4	0.099

Table S3. The stratification of the crude incidence rate based on the diagnosis of the type of traumatic brain injury.

Year	Age	2008	2009	2010	2011	2012	2013	2014	2015	2016	2017	*p* for Trend
Concussion	0–4	1538.8	1662.2	1683.5	1568.2	1553.9	1490.7	1324.5	1218.0	1234.8	1202.4	<0.001
	5–9	716.5	770.0	810.0	782.6	793.9	765.6	720.8	678.1	653.7	665.7	<0.001
Intracranial injury	0–4	293.8	272.9	257.3	223.4	183.1	185.3	157.7	132.4	129.7	114.1	<0.001
	5–9	137.4	132.0	119.7	110.5	92.9	86.1	76.2	66.5	70.2	65.5	<0.001
